# Stillbirth and Associated Factors Among Women Who Gave Birth at Hiwot Fana Specialized University Hospital, Harar, Eastern Ethiopia

**DOI:** 10.3389/fped.2022.820308

**Published:** 2022-05-12

**Authors:** Seble Mengistu, Adera Debella, Teshale Mulatu, Firehiwot Mesfin, Kababa Temesgen Danusa, Merga Dheresa

**Affiliations:** ^1^Hiwot Fana Specialized University Hospital, College of Health and Medical Science, Haramaya University, Dire Dawa, Ethiopia; ^2^School of Nursing and Midwifery, College of Health and Medical Science, Haramaya University, Dire Dawa, Ethiopia; ^3^School of Public Health, College of Health and Medical Science, Haramaya University, Dire Dawa, Ethiopia; ^4^Department of Midwifery, College of Medicine and Health Sciences, Ambo University, Ambo, Ethiopia

**Keywords:** stillbirth, associated factors, women, birth, Eastern Ethiopia

## Abstract

**Background:**

Stillbirth, which accounts for half of all the perinatal mortality, is not counted on policy, program, and investment agendas around the globe. It has been underestimated public health burden, particularly in developing countries. Ethiopia is among the top countries with a large prevalence of stillbirth in the world. However, there is a dearth of study on the current magnitude of stillbirth in the study area. Therefore, this study intended to assess the prevalence of stillbirth and its associated factors to bridge the gap.

**Methods:**

A hospital-based retrospective study was conducted from 1 to 28 February 2019 and data were collected by reviewing the chart records of all the women who gave birth in the past 2 years (January 2016 to December 2018) at Hiwot Fana Specialized University Hospital. Data were entered into EpiData version 4.2.0.0 software and transported to SPSS version 23 for analysis. Descriptive statistics such as frequency, mean, and SDs were generated. Determinants of stillbirth were analyzed using a binary logistic regression and presented by adjusted odds ratio (AOR) with a 95% CI.

**Results:**

The prevalence of stillbirth was 14.5% (95% CI: 11.7%, 17.6%). Low birth weight (AOR = 2.42, 95% CI: 1.23–4.76), prematurity (AOR = 2.10, 95% CI: 1.10–4.01), premature rupture of membranes (AOR = 2.08, 95% CI: 1.14–3.77), antepartum hemorrhage (AOR = 3.33, 95% CI: 1.66–6.67), obstructed labor (AOR = 2.87, 95% CI: 1.48–5.56), and preeclampsia (AOR = 2.91, 95% CI: 1.28–6.62) were an independently associated with stillbirth.

**Conclusion:**

The prevalence of stillbirth in this study was high. Low birth weight, preterm birth, premature rupture of membranes, antepartum hemorrhage, obstructed labor, and preeclampsia were independently associated with a stillbirth. Therefore, much study is needed involving different stakeholders to reduce stillbirths by improving the health status of women through the provision of quality maternal care including referral systems.

## Background

Stillbirth is defined as a fetus born with no signs of life at or after 28 weeks of gestation. It is an adverse pregnancy outcome and tragedy, which causes a substantial psychological burden to the mother and the families (1, 2).

Stillbirths are largely absent from global indicators of health, which underestimate its extent of public health importance. Globally, an estimated 2.0 million babies were stillbirth at 28 weeks or more of gestation, with a global stillbirth rate of 13.9 stillbirths per 1,000 total births. Stillbirth rates in 2019 varied widely across regions, from 22.8 stillbirths per 1,000 total births in the west and central Africa to 2.9 (2.7–3.0) in Western Europe. After west and central Africa, eastern and southern Africa and South Asia had the second and third highest stillbirth rates in 2019. For the remaining 81 countries, we found no decrease in the stillbirth rate since 2000. Of these countries, 34 were in sub-Saharan Africa, 16 were in East Asia and the Pacific, and 15 were in Latin America and the Caribbean. It is estimated that in these locations, about half of all the stillbirths occur in the intrapartum period (3).

Every newborn action plan to end preventable deaths has a set stillbirth target of 12 per 1,000 births or less by 2030 (4). However, the reduction of stillbirths has lagged behind that of maternal and under-five child mortality; from 2000 to 2015, stillbirth rate was reduced from 24.7 to 18.4 per 1,000 births (5). Progress in decreasing of this rate has been sluggish and at the present rate, 160 years will elapse before a pregnant woman in Africa has the similar opportunity of her fetus being born alive as a woman in a developed country today (1, 6).

Ten countries accounted for 66% of the world's stillbirths, while Ethiopia ranked seventh among the top ten countries (7). Evidence from the community and facility-based studies portrayed the stillbirth rate 19.6–87 per 1,000 births in Ethiopia (8, 9).

Even though the number of institutional birth is increasing, more avoidable perinatal mortality and morbidity are occurring in health facilities. This can be explained by the quality care provided at the health facility at the time of delivery. Improving the quality of care in health facilities is imperative to end preventable mortality and morbidity among mothers and newborns. On the other hand, stillbirths are strongly linked to adverse social and economic determinants. Factors related to the health system alone cannot address entirely the problem of stillbirths. The common predictors were attributed to personal behaviors, socioeconomic factors, reproductive health, environmental health, and health care infrastructures (10–13).

The majority of stillbirths and neonatal deaths are avoidable with quality healthcare during pregnancy and childbirth. Despite numerous strategies and interventions, the prevention of stillbirth remains neglected in many countries. Almost all the offspring who are stillborn and half of all the neonatal losses are not documented in a birth or death record and, thus, have never been registered, reported, or investigated by the health system (2, 14).

The government of Ethiopia had been implementing different effective programs to improve maternal and child health through capacity building and strengthening the healthcare delivery system to improve the quality of service during pregnancy such as antenatal care and safe delivery practice (15). Despite the governmental and non-governmental efforts to reduce the rate of stillbirth, prevention of stillbirth has remained largely unaddressed. In addition, stillbirths are not registered systematically and records lack consistency in low-income countries such as Ethiopia, which lead to underestimation of stillbirths. Assessment of the burden of stillbirth and associated factors in healthcare setup is important for developing strategies to prevent or reduce stillbirths. Therefore, this study aimed to bridge the gap by assessing the prevalence of stillbirth and its associated factors among women who gave birth in Hiwot Fana Specialized University Hospital, Eastern Ethiopia.

## Methods and Materials

### Study Area and Period

This study was conducted at Hiwot Fana Specialized University Hospital. This hospital is located in the Harari Regional State, which is 526 km away from Addis Ababa to the East. Currently, the hospital serves about 5.8 million people from the Harari region, Dire Dawa Administration, Oromia region, and Somali region. It serves as a teaching center of Eastern Ethiopia and delivers full-service healthcare to the community, including internal medicine, surgery, gynecology and obstetrics, TB/HIV, and other healthcare services (16, 17). This study was conducted from 1 to 28 February 2019 (by reviewing charts from January 2016 to December 2018).

### Study Design and Population

A facility-based retrospective cross-sectional study that involved chart review was conducted. Record of all the women delivered with a gestational age of 28 weeks or more and birth weight ≥ 1,000 g was included in this study, while a record with an incomplete document, missing pertinent variables was excluded.

### Sample Size and Sampling Techniques

The sample size was determined by using double population proportion formula considering factors that are significantly associated with the outcome variable at *p* < 0.05, a two-sided confidence level of 95%, the margin of error of 5%, power of 80%, and the ratio of exposed to unexposed 1:1 using Epi-calc statistical software. Taking antenatal care (ANC) to follow-up as exposure variable (no ANC visits = 9.1%, having ≥ one ANC visits = 2.9%) (18) and then by adding 10% non-response rate, the final required sample size was 574. Records were selected by using systematic random sampling. The delivery registration book was used as the sampling frame. Records were retrieved using medical record numbers found from the labor ward, maternity ward, and operation room log books. Then, patient's card was collected from the archive room of the hospital. Only those maternal records found within the archive of this hospital and who gave birth (delivered) at this hospital over 2 years (January 2016 to December 2018) were included in this study.

### Data Collection Tool and Procedure

Data were collected by using a structured and pretested abstraction checklist adapted from similar studies conducted on the topic. A 2-day training was given to both the data collectors and the supervisors regarding the objective of this study, data collection tool, and ways of data collection. The tool was pretested on 5% of the sample size in Jugol Hospital before the actual data collection. Correction and modifications were made to the tool based on the result of the pretest. All the maternal records found from logbooks and cards were reviewed to obtain any information pertinent to the mother and newborn, including obstetric complications, maternal medical conditions, fetal conditions, ultrasound findings and reports, and other cases, which need a diagnosis for confirmation. Stillbirth was ascertained from the mother's medical records confirmed with professionals. Collected data were checked for accuracy and completeness daily.

### Data Processing and Analysis

After the data had been checked for completeness and internal consistency, coded and double entered into EpiData version 4.2.0 and exported to SPSS version 23 for further data cleaning and analysis. Frequency was generated to check for any missing values. Prevalence of stillbirth was presented using proportion with 95% CI and a binary logistic regression was used to see the association between the outcome variable and each independent variable.

All the variables with a *p*-value ≤ 0.2 presented in crude odds ratio (COR) were taken into the multivariate logistic regression analysis model to control for all the possible confounders. The Hosmer–Lemeshow goodness-of-fit model was used to assess model fitness and the results showed that model is well fitted to the multivariate logistic regression analysis model (chi-squared value of test = 6.43, *p*-value = 0.101). Finally, the results of the multivariate logistic regression analysis were presented in an adjusted odds ratio (AOR) with 95% CIs. The level of statistical significance was declared at a *p*-value < 0.05.

## Results

### Sociodemographic Characteristics

A total of 557 delivered women's charts were reviewed, yielding a response rate of 97.3%. The mean age of the participants was 25.97 (±5.399 SD) years. Three hundred twenty-seven (58.7%) of participants were between 20 and 34 years old. Concerning their marital status and residency, 548 (98.4%) of them were married and 302 (54.2%) of respondents were rural inhabitants (Table 1).

**Table 1:** Sociodemographic characteristics of study participants (*n* = 557), Hiwot Fana Specialized University Hospital, Harar, Eastern Ethiopia, 2018/2019.

**Variables (*n* = 557)**	**Frequency**	**Percentage (%)**
Age		
<20	163	29.3
20–34	327	58.7
35+	67	12.0
Marital status		
Married	548	98.4
Other^*^	9	1.6
Place of residence		
Urban	255	45.8
Rural	302	54.2

Other^*^ = single, divorced, widowed.

### Obstetrics-Related Characteristics

Regarding the parity of participants, 210 (37.7%) were multiparous and 201 (36.1%) were primiparous, respectively. In addition, 43 (7.7%) had a history of abortion, while 32 (5.7%) had a history of stillbirth (Table 2).

**Table 2:** Obstetrics condition of study participants (*n* = 557), Hiwot Fana Specialized University Hospital, Harar, Eastern Ethiopia, 2018/2019.

**Variables**	**Stillbirth**		**Frequency (%)**
	**Yes (%)**	**No (%)**	
ANC visit			
Yes	46 (56.8)	340 (71.5)	386 (69.3)
No	35 (43.2)	136 (28.57)	171 (30.7)
Number of ANC visit *n* = 386)			
1	10 (21.7)	31 (9.12)	41 (10.6)
2–3	30 (65.2)	225 (66.2)	255 (66.1)
≥4	6 (13)	84 (24.7)	90 (23.3)
Parity			
Primiparous	25 (30.9)	176 (37)	201 (36.1)
Multiparous	21 (25.9)	189 (39.7)	210 (37.7)
Grand multiparous	35 (43.2)	111 (23.3)	146 (26.2)
Obstructed labor			
Yes	23 (28.4)	65 (13.7)	88 (15.8)
No	58 (71.6)	411 (86.3)	469 (84.2)
PROM			
Yes	32 (39.5)	136 (28.6)	168 (30.2)
No	49 (60.5)	340 (71.4)	389 (69.8)
APH			
Yes	31(38.3)	54 (11.3)	85 (15.3)
No	50 (61.7)	422 (88.7)	472 (84.7)
Cord prolapsed			
Yes	2 (2.47)	10 (2.1)	12 (2.2)
No	79 (97.5)	466 (97.9)	545 (97.8)
Pregnancy status			
Wanted	75 ()	458 (82.2)	533 (95.7)
Unwanted	6 (7.41)	18 (3.78)	24 (4.3)
Fetal condition at admission			
Alive	33 (40.7)	467 (98.1)	500 (89.8)
Dead	46 (56.8)	6 (1.26)	52 (9.3)
In distress	2 (2.47)	3 (.63)	5 (0.9)
History of abortion			
Yes	4 (4.94)	39 (8.19)	43 (7.7)
No	77 (95.1)	437 (91.8)	514 (92.3)
History of stillbirth			
Yes	12 (14.8)	20 (4.2)	32 (5.7)
No	69 (85.2)	456 (95.8)	525 (94.3)
Labor onset			
Spontaneous	52 (64.2)	420 (88.2)	472 (84.7)
Induced	29 (35.8)	56 (11.8)	85 (15.3)
Mode of delivery			
SVD	45 (55.6)	319 (67)	364 (65.3)
Caesarian section	28 (34.6)	129 (27.1)	157 (28.2)
^*^Others	8 (9.9)	28 (5.9)	36 (6.5)

*ANC, Antenatal care; APH, Antepartum hemorrhage; PROM, Premature rupture of membrane; SVD, Spontaneous vaginal delivery;*

* *Others, Instrunmental and destructive deliveries*.

### Medical Condition of the Study Participants

Regarding the medical condition of the mothers, a total of 180 (32.3%) mothers had a history of anemia. Concerning hypertensive disorder of pregnancy, 43 (7.7%) and 15 (2.7%) of the mothers had a history of preeclampsia and eclampsia (Table 3).

**Table 3:** Medical condition of study participants (*n* = 557), Hiwot Fana Specialized University Hospital, Harar, Eastern Ethiopia, 2018/2019.

**Variables**	**Stillbirth**		**Frequency (%)**
	**Yes (%)**	**No (%)**	
Anemia			
Yes	47 (58)	133 (28)	180 (32.3)
No	34 (42)	343 (72)	377 (67.7)
Gestational DM			
Yes	1 (1.23)	5 (1.1)	6 (1.1)
No	80 (98.8)	471 (98.9)	551 (98.9)
Preeclamsia			
Yes	12 (14.8)	31 (6.5)	43 (7.7)
No	69 (85.2)	445 (93)	514 (92.3)
Eclampsia			
Yes	5 (6.17)	10 (2.1)	15 (2.7)
No	76 (93.8)	466 (98)	542 (97.3)
UTI			
Yes	7 (8.64)	35 (7.4)	42 (7.5)
No	74 (91.4)	441 (93)	515 (92.5)

*DM, Diabetes mellitus; UTI, urinary tract infection*.

### Fetal Condition-Related Characteristics

Of the total included records, 17 (3.1%) of the mother had given birth to a newborn with congenital anomaly, while 123 (22.1%) were low birth weight (Table 4).

**Table 4:** Fetal-related characteristics of study participants (*n* = 557), Hiwot Fana Specialized University Hospital, Harar, Eastern Ethiopia, 2018/2019.

**Variables**	**Stillbirth**		**Frequency (%)**
	**Yes (%)**	**No (%)**	
Congenital anomaly			
Yes	8 (9.8)	9 (1.89)	17 (3.1)
No	73 (90.1)	467 (98.1)	540 (96.9)
Weight at birth			
Normal	41 (50.6)	393 (82.6)	434 (77.9)
LBW	40 (49.4)	83 (17.4)	123 (22.1)
GA at birth			
Term	31 (38.3)	326 (68.5)	357 (64.1)
Preterm	50 (61.7)	150 (31.5)	200 (35.9)
IUGR			
Yes	2 (2.47)	6 (1.26)	8 (1.5)
No	79 (97.5)	470 (84.3)	549 (98.5)
Malpresentation			
Yes	14 (17.3)	68 (14.3)	82 (14.7)
No	67 (82.7)	408 (85.7)	475 (85.3)
Newborn sex			
Male	60 (74.1)	297 (62.4)	357 (64.1)
Female	21 (25.9)	179 (37.6)	200 (35.9)

*LBW, Low birth weight; GA, Gestational age; IUGR, Intrauterine growth restriction*.

### Prevalence of Stillbirth

**Figure 1:**
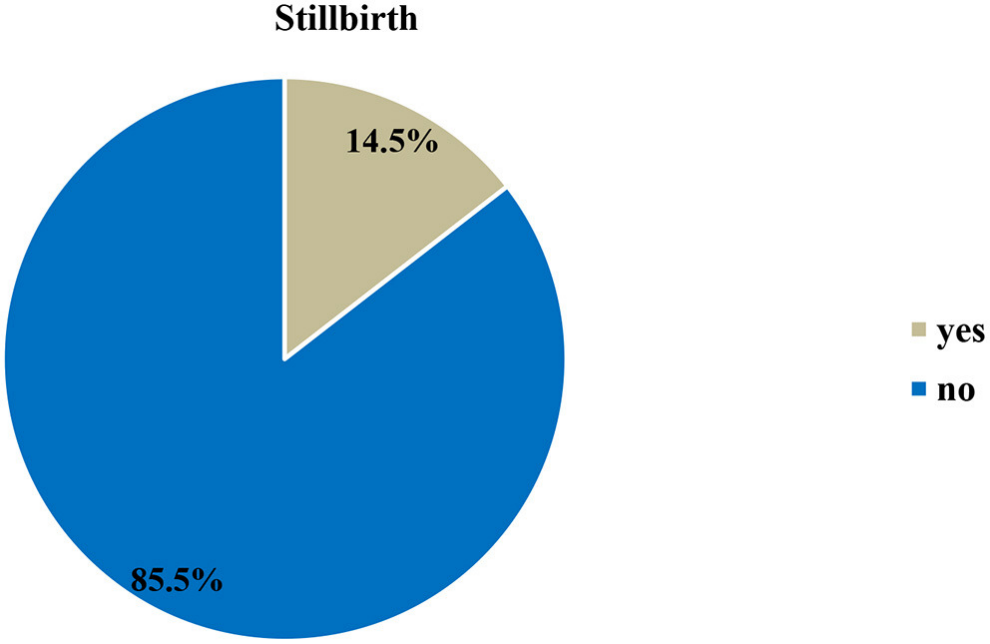
Prevalence of stillbirth at Hiwot Fana Specialized University Hospital, Harar, Eastern Ethiopia, 2018/2019 (*n* = 557).

The rate of stillbirth in this study was 14.5% (95% CI: 11.7%, 17.6%) (Figure 1).

### Factors Associated With Stillbirth

The relationship between each independent variable and the dependent variable was separately analyzed. In the bivariate analysis, place of residence, ANC visit, history of anemia, birth weight, gestational age at birth, history of premature rupture of membranes (PROM), history of antepartum hemorrhage (APH), obstructed labor, eclampsia, and preeclampsia were found to be associated factors with a stillbirth at *p*-value < 0.2. After adjusting for other variables, birth weight, gestational age at birth, history of PROM, history of APH, obstructed labor, and preeclampsia maintained their association with stillbirth in the multivariate model. Low birth weight fetuses were 2.42 times more likely to be a stillbirth (AOR = 2.42, 95% CI: 1.23–4.76). Mothers with preterm delivery were 2.1 times more likely to have a stillbirth than mothers who delivered at term delivery (AOR = 2.10, 95% CI: 1.10–4.01). Mothers who had a history of APH were 3.33 times (AOR = 3.33, 95% CI: 1.66–6.67) more likely to have stillbirth as compared to women who had no history of APH. Mothers who had a history of preeclampsia were 2.91 times (AOR = 2.91, 95% CI: 1.28–6.62) more likely to have stillbirth as compared to women who had no preeclampsia (Table 5).

**Table 5:** The multivariate analysis of factors associated with a stillbirth at Hiwot Fana Specialized University Hospital, Harar, Eastern Ethiopia, 2018/2019 (*n* = 557).

**Variables**	**Stillbirth**		**COR (95%CI)**	**AOR (95%CI)**
	**Yes (%)**	**No (%)**		
Anemia				
Yes	47 (58)	133 (28)	3.56 (2.19–5.78)	1.57 (0.84–2.93)
No	34 (42)	343 (72)	1	1
Residence				
Rural	55 (67.9)	247 (51.8)	1.96 (1.19–3.23)	1.06 (0.59–1.93)
Urban	26 (32.1)	229 (48.1)	1	1
Weight at birth				
LBW	40 (49.4)	83 (17.4)	4.61 (2.81–7.58)	2.42 (1.23–4.76)^*^
Normal	41 (50.6)	393 (82.6)	1	1
GA at birth				
Preterm	50 (61.7)	150 (31.5)	3.50 (2.15–5.71)	2.10 (1.10–4.01)^*^
Term	31 (38.3)	326 (68.5)	1	1
PROM				
Yes	32 (39.5)	136 (28.6)	1.63 (1.01–2.65)	2.08 (1.14–3.77)^*^
No	49 (60.5)	340 (71.4)	1	1
APH				
Yes	31 (38.3)	54 (11.3)	4.84 (2.85–8.23)	3.33 (1.66–6.67)^*^
No	50 (61.7)	422 (88.7)	1	1
Obstructed labor				
Yes	23 (28.4)	65 (13.7)	2.50 (1.44–4.34)	2.87 (1.48–5.56)^*^
No	58 (71.6)	411 (86.3)	1	1
ANC visit				
Yes	46 (56.8)	340 (71.5)	1	1
No	35 (43.2)	136 (28.57)	1.90 (1.17–3.08)	1.19 (0.67–2.10)
Eclampsia				
Yes	5 (6.17)	10 (2.1)	3.06 (1.02–9.21)	2.34 (0.64–8.50)
No	76 (93.8)	466 (98)	1	1
Preeclamsia				
Yes	12 (14.8)	31 (6.5)	2.49 (1.22–5.09)	2.91 (1.28–6.62)^*^
No	69 (85.2)	445 (93)	1	1

* *AOR significant at P-value < 0.05*.

*COR, Crude odd ratio; AOR, Adjusted odd ratio*.

*Significant at P-value < 0.2*.

*1.00 = reference*.

*ANC, Antenatal care; APH, Antepartum hemorrhage; PROM, Premature rupture of membrane*.

## Discussion

This study assessed the prevalence of stillbirth and its associated factors among women who gave birth at Hiwot Fana Specialized University Hospital in Harar town, Eastern Ethiopia. The prevalence of stillbirth in this study was 14.5% (95% CI: 11.7%, 17.6%). Low birth weight, gestational age at birth, history of PROM, history of APH, obstructed labor, and preeclampsia were the factors significantly associated with a stillbirth.

The proportion of stillbirth in this study was considerably higher with respect to global stillbirth rate estimates of 13.9/1,000 births in 2019 (3) and the United Nations (UN) stillbirth rate recommendation of Every Newborn Action Plan to end preventable causes of stillbirth, which recommends the stillbirth rate to be reduced below 12/1,000 births (4, 5). This high prevalence of stillbirths could be attributed to poor referral systems, poor transport facilities, and long distances to this referral hospital, which are all significant in this study setting. The magnitude of stillbirth in this study is also high when compared to the studies conducted in Africa, Nigeria (4.8%), Hossana (8.6%), and Jimma (8%) in Ethiopia (19–21). The discrepancy might be due to the setting difference where this study was conducted; sociocultural differences and sample size variation may also attributed to the discrepancy.

Premature rupture of membranes was one of the associated factors of stillbirth in this study. Mothers who had come across premature rapture of membranes had 2-fold odds of stillbirth than mothers who had no premature rupture of membranes. This study is in line with the study conducted in Nigeria and Bahirdar, Northwest Ethiopia (19, 22). This is probably a fetus born from a mother with premature rupture of membranes might have faced perinatal asphyxia, which grounds fetal death. Hence, screening and follow-up for a mother who had a history of PROM during pregnancy and labor are essential to reduce the risk of stillbirths.

In addition, gestational age was found to be associated with stillbirth. Those delivered before 37 weeks of gestational age were twice as likely to suffer a stillbirth compared to term delivery. This finding is supported with evidence from Tanzania and Jimma that showed being preterm as a risk for stillbirth (21, 23). This connotation might be due to the fact that premature newborns had less time to grow in the mother's uterus with minimal lung maturity and they are at risk of being asphyxiated and distressed. Hence, mothers who were diagnosed with preterm labor should be monitored or followed strictly to prevent stillbirth related to prematurity and its complication.

On the other hand, low birth weight fetuses were about 2.4 times more likely to be born as stillbirth compared to normal birth weight fetuses. This finding is in line with the evidence from Nepal, Addis Ababa, and Gondar (8, 24, 25). This could be due to the fact that low birth weight fetuses are more likely to be immature and could not tolerate intrauterine asphyxia because of different physiological reasons. So, early screening of problems that results in low birth weight is essential to minimize the risk of stillbirth related to low birth weight and its complication.

Furthermore, APH, preeclampsia, and obstructed labor were found to be significantly associated with stillbirth. Similarly, a study from Taiwan and Gondar also showed that hypertensive disorders of pregnancy, obstructed labor, uterine rupture, and cord accident combined were significantly associated with stillbirth. This is probably due to preeclampsia, which will complicate the fetal outcome through placental abruption, leading to the death of the fetus in the uterus. In addition, obstructed labor also contributes to stillbirth through different complications such as sepsis, fetal distress, and uterine rupture (8, 26). In this study, mothers who had a history of APH were 3.3 times more likely to have stillbirth compared to women who had not a history of APH. This finding is in line with other studies done in Nigeria and Nepal (27, 28). This may be due to the fact that antepartum hemorrhage during pregnancy may result due to placental abnormality, which may cause placental oxygen and nutrient insufficiency to the fetus (29). In addition, excess bleeding from APH may result in anemia and decreased placental perfusion, which, in turn, results in intrauterine hypoxia, development of infection, and premature delivery that increase the risk of stillbirth. This implies that appropriate management for APH or causes of APH is crucial to minimize the risk of stillbirth.

## Limitation of This Study

Since it was conducted only in a single facility, it may not be generalizable for the total communities and health facilities in the study area. Additionally, this study assessed stillbirth through retrospective record review; the record may not necessarily reflect those essential predisposing factors that lead women to develop stillbirth.

Again the causes of stillbirths were not identified as antepartum or intrapartum. This is mainly due to incomplete documentation, as many of the charts have no specific information on the causes and types of stillbirths. No histopathological and autopsy examination was done to determine the causes of deaths for stillbirths because these diagnostic modalities were not feasible in our setting due to lack of infrastructures, resources, and accessibility of the services for community at large.

## Conclusion

Generally, the prevalence of stillbirth in this study was high compared to other studies. Low birth weight, prematurity, history of premature rupture of membranes, history of antepartum hemorrhage, obstructed labor, and preeclampsia were an independent risk factors found to be associated with stillbirth. Since most of the factors are preventable, prenatal risk identification for early diagnosis and treatment and prompt referral to nearby hospitals are essential for reducing delays in the provision of appropriate care.

## Data Availability Statement

The raw data supporting the conclusions of this article will be made available by the authors, without undue reservation.

## Ethics Statement

Ethical approval was obtained from the Institutional Health Research Ethics Review Committee (IHRERC) of the College of Health and Medical Sciences, Haramaya University. The patients/participants provided their written informed consent to participate in this study.

## Author Contributions

SM, TM, AD, FM, MD, and KD conceived the study and were involved in the study design, reviewed the article, analysis, report writing, and drafted the manuscript. All the authors have read and approved the final version of the manuscript.

## Funding

This study was funded by Haramaya University. The funding organization has no role in designing the study, data collection, analysis, and its interpretation, protocol writing, or submission.

## Conflict of Interest

The authors declare that the research was conducted in the absence of any commercial or financial relationships that could be construed as a potential conflict of interest.

## Publisher's Note

All claims expressed in this article are solely those of the authors and do not necessarily represent those of their affiliated organizations, or those of the publisher, the editors and the reviewers. Any product that may be evaluated in this article, or claim that may be made by its manufacturer, is not guaranteed or endorsed by the publisher.
